# Elucidating the Potential Mechanisms Underlying Distraction Spinal Cord Injury-Associated Neuroinflammation and Apoptosis

**DOI:** 10.3389/fcell.2022.839313

**Published:** 2022-02-21

**Authors:** Bo Han, Weishi Liang, Yong Hai, Yuzeng Liu, Yuxiang Chen, Hongtao Ding, Jincai Yang, Peng Yin

**Affiliations:** Department of Orthopedic Surgery, Beijing Chaoyang Hospital, Capital Medical University, Beijing, China

**Keywords:** distraction spinal cord injury, neuroinflammation, apoptosis, inflammatory cytokines, porcine model

## Abstract

The incidence of distraction spinal cord injury (DSCI), which results from spinal cord ischemia due to vascular compromise and spinal cord tract disturbances, remains high. Furthermore, because no ideal animal model that mimics DSCI in clinical settings is available thus far, the related molecular mechanisms underlying DSCI remain unclear. Thus, this study aimed to establish a porcine model of DSCI and investigate the neuroinflammation and apoptosis mechanisms in these pigs. Before surgery, all pigs were randomly divided into three groups: sham group, osteotomy surgery only; the incomplete distraction spinal cord injury (IDSCI) and complete distraction spinal cord injury (CDSCI) group, osteotomy plus DSCI surgery with a motor-evoked potential (MEP) amplitude decreased by approximately 75% and 100%, respectively. After surgery, modified Tarlov scoring and MRC muscle strength scoring were used to evaluate neurologic function in each group. We observed the distracted spinal cord using MRI, and then all pigs were sacrificed. Inflammatory cytokine levels in the spinal cord and cerebrospinal fluid (CSF) were also analyzed. We used immunofluorescence staining to assess the neuronal and microglial structure and function and astrocyte hyperplasia in the central DSCI lesions (T15). Western blotting was used to determine the expression of apoptosis-related proteins. Results showed that the modified Tarlov scoring and muscle strength decreased significantly in the two DSCI groups. T2-MRI showed a relative enhancement at the center of the DSCI lesions. H&E and Lxol fast blue staining revealed that spinal cord distraction destroyed the normal structure of spinal cord tissues and nerve fiber tracts, exacerbating inflammatory cell infiltration, hyperemia, and edema. The IL-1β, IL-6, and TNF-α levels increased in the spinal cord and CSF following DSCI. Immunofluorescence staining results indicated the GFAP, Iba-1 expression increased following DSCI, whereas the NeuN expression reduced. Moreover, DSCI promoted the protein expression of P53, Bcl-2-associated X protein (Bax), and Caspase-3 in the spinal cord tissues, whereas it reduced the Bcl-2 expression. This study successfully established a porcine DSCI model that closely mimics DSCI in clinical settings, and clarified the mechanisms underlying DSCI-associated neuroinflammation and apoptosis; thus, our findings highlight potential DSCI-treatment strategies for further establishing suitable drug therapies.

## 1 Introduction

Spinal cord injury (SCI), caused by contusion, dislocation, or distraction due to a sequential combination of primary and secondary injury (Chen et al., 2016), has devastating consequences for the physical, economic, and mental health of the patients and their caregivers (Ahuja et al., 2017). The changes in secondary degeneration are altered by the primary injury that should be highlighted both clinically and pre-clinically (Mattucci et al., 2021). Given population growth, there were 0.93 million new cases of SCI worldwide with age-standardized incidence rates of 13 per 100000, in 2016; this is expected to increase (Collaborators, 2019). Transection and contusion injury paradigms have been widely used in preclinical studies of SCI (Kjell and Olson, 2016). However, other injuries, such as spinal cord stretching from distraction injuries, contusion from vertebral burst fracture, and shearing from fracture-dislocation, also occur frequently in clinical settings (McDonald and Sadowsky, 2002). It has been reported that different neuroprotective strategies may be required for treating distinct clinically relevant SCIs.

Distraction spinal cord injury (DSCI) is thought to be caused by spinal cord ischemia due to vascular compromise and direct traction-induced spinal cord tract disturbances (Seyal and Mull, 2002). Currently, the main reason for SCI during spinal deformity correction is distraction injury (Hamilton et al., 2011; Charosky et al., 2012). With the application of the growing rod (Helenius et al., 2018) and the appearance of vertebral column resection osteotomy (Yang et al., 2016) in patients with severe deformities, the incidence of DSCI remains higher than that in the past few decades. Since DSCI was first reported in the 1970s (Martin et al., 1971; Fried, 1974), several clinical studies have focused on stretching-induced SCI and established relevant animal models using mice and rabbits (Wu et al., 2016; Bell et al., 2017; Tica et al., 2018; Guo et al., 2019). However, currently, a suitable DSCI model that mimics clinical DSCI and can be used to study the cytological and molecular mechanisms underlying DSCI is still unavailable.

The neuroinflammatory and apoptosis process are thought to play a pivotal role in secondary injury after SCI (Hohlfeld et al., 2007; Alizadeh et al., 2019). This process is characterized by acute microglial activation, followed by the delayed activation of astrocytes that exacerbates tissue damage by releasing reactive oxygen species, pro-inflammatory cytokines/chemokines, proteases, and lysosome enzymes (Ren et al., 2018). Inflammatory cytokines, as direct mediators, affect the prognosis of spinal cord injury to different degrees (Hu et al., 2016). One of the main pathological features of SCI is neuronal apoptosis (Huang et al., 2021), a kind of energy-dependent programmed death, which can be divided into exogenous and endogenous pathways according to triggering mechanisms (Ren et al., 2019). Apoptosis occurs within a few hours after primary SCI and reaches the peak within several days (Beattie et al., 2000). With the deepening of research, studies on SCI have paid more attention to the Bcl-2/Bcl-2-associated X protein (Bax)/Caspase-3 pathway in apoptosis gradually (Li et al., 2015). The expression of Bax and Caspase-3 and activated Bcl-2 can well reflect the regulation mechanism regarding apoptosis. In the present study, we successfully established a porcine DSCI model mimicking clinical DSCI, and clarified the role of microglial and astrocyte neuroinflammation and apoptosis in DSCI, highlighting potential strategies for DSCI treatment.

## 2 Materials and Methods

### 2.1 Animal Caring

Nine newly purchased experimental Bama pigs (3-month-old, 11.40 ± 1.68 kg, China) were adaptatively fed for 1 week. All pigs were housed and underwent experiments in Large Animal Laboratory, Center of Experimental Animals of Capital Medical University, and kept in a humidity- and temperature-controlled environment with a 12-h light-dark cycle. Animal experiments complied with the Guide for the Care and Use of Laboratory Animals published by the National Institutes of Health (NIH Publication No. 8523, revised 2011, United States). All experimental projects and protocols were approved by the Medical Ethics Committee of Capital Medical University (AEEI-2019-098).

### 2.2 Grouping and Neuromonitoring

**FIGURE 1:**
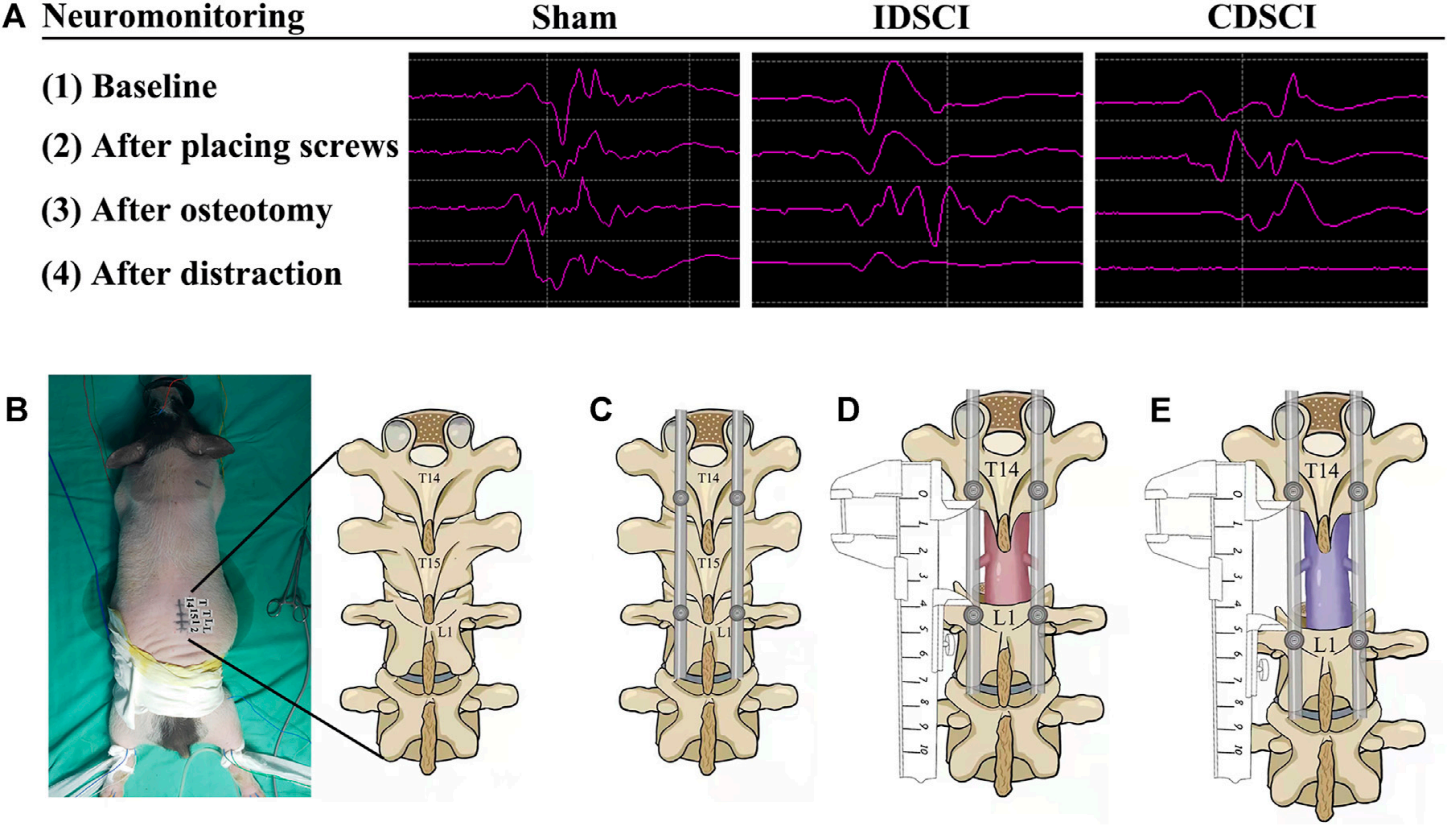
Intraoperative MEP changes in pigs of three different groups, and the schematic diagram of surgical procedure of the porcine DSCI model establishment. **(A)** Representative images (baseline, after placing screws, after osteotomy, after distraction) of MEP responses were recorded in the gastrocnemius muscles of porcine hind limbs during DSCI surgery. **(B)** A midline longitudinal incision was made, and the laminae from T14, T15, and L1 were exposed with coagulation cautery throughout. **(C)** Pedicle screws and rods were used to fix T14 and L1 temporarily. **(D)** Global column osteotomy was performed at T15 under the protection of a root retractor, and the T15 vertebral body and adjacent discs were completely resected. **(E)** A spreader was used to pull the spine gradually, and the distance between the T14 and L1 pedicle screws was measured before and after distraction.

Prior to surgery, all pigs were randomly divided into three groups (Figure 1A): sham group (*n* = 3), osteotomy only with normal motor evoked potential (MEP); incomplete distraction spinal cord injury (IDSCI) group (*n* = 3): osteotomy with DSCI, MEP amplitude decreased by approximately 75%; and complete distraction spinal cord injury (CDSCI) group (*n* = 3): osteotomy with DSCI, MEP amplitude decreased to 0. Any abnormal MEP signals compared with the baseline amplitude were considered to imply accidental iatrogenic SCI, and we excluded these animals from the experimental group. The spinal cord-evoked potential was monitored using an electrophysiological monitor (Cadwell, United States) during the operation. The stimulating electrodes were placed in the C3-C4 cortical motor area, and the single stimulation intensity was 100–200 V. Needle-like recording and reference electrodes were placed on the gastrocnemius muscles of both hind limbs, and the distance between the two needle electrodes was approximately 1.5 cm. The compound muscle action potential induced by a single electrical stimulus was recorded. MEP was recorded and a baseline was established after successful anesthesia. MEP was monitored throughout the spinal cord during placing screws, osteotomy, distraction (Figure 1A). Applying longitudinal distraction force to the vertebra to stretch the spinal cord until the MEP amplitude decreased by approximately 75% or 100%, and maintained the amplitude changes for 10 min. Animals were sacrificed 7 days after magnetic resonance imaging (MRI), and spinal cords were collected and processed for histological, molecular and biochemical analyses.

### 2.3 Generation of the Porcine Distraction Spinal Cord Injury Model

As shown in Figures 1B–E, the schematic of DSCI surgical procedure was displayed. A midline longitudinal incision extending from T13 to L2 was made, and the laminae from T14, T15, and L1 were exposed with coagulation cautery; 4.0 mm*25 mm short coccygeal pedicle screws (Weigao Orthopedic Materials Co., Ltd, China) were infixed into the vertebrae of T14 and L1. SINO rods of suitable length (Weigao Orthopedic Materials Co., Ltd, China) were installed on the pedicle screws on the right and left sides for temporary fixation. Global column osteotomy was performed at T15 under the protection of a root retractor, and the T15 vertebral body and adjacent discs were completely resected. A spreader was used to pull the spine at 1 mm intervals gradually. The distraction operation was performed according to MEP changes. The distance between the pedicle screws of T14 and L1 was measured before and after the DSCI procedure. After the muscles and skin were sutured, rigorous wound care was performed every 3 days. 1.5 g Cefuroxime sodium was administered for 3 days after surgery to prevent incision infection. Additionally, the bladder of each pig was manually emptied twice per day, and rigorous care of the perineum was taken to prevent urinary infection.

### 2.4 Anesthesia Monitoring and Wake-Up Test

The animals were made to fast for 24 h before anesthesia. We performed the operation under anesthesia with a solution of 3% pentobarbital sodium injected intramuscularly. Subsequently, the pigs were treated with an oxygen mask and isoflurane inhalation anesthesia. Each pig was intubated with an endotracheal tube and maintained under inhalation anesthesia without a muscle relaxant (Kaiser et al., 2006). The depth of anesthesia was monitored using pain and corneal reflex tests. The depth of anesthesia was adjusted at all times to ensure that the pig breathed smoothly to prevent convulsions during image acquisition. Three vital signs, including rectal temperature, respiratory rate, and heart rate, were continuously monitored using a multifunctional bedside physiological monitor (PVM-2701; Nihon Kohden Corporation, Tokyo, Japan).

At the end of the DSCI model generation surgery, a wake-up test was carried out to prevent any false-negative or false-positive MEP signals from occurring. Approximately 30 min after osteotomy stabilization in animal model generation surgery, the pigs were awakened from anesthesia. We tested and observed lower extremity movements and sensory responses to mechanical stimulation.

### 2.5 Postoperative Neurologic Function Assessment

Neurologic function assessment for pigs was performed at 1, 3, and 7 days postoperatively. According to the modified Tarlov score, we assessed pairs of hind limbs of the pigs. The modified Tarlov scoring was as follow (Assina et al., 2008): 0 point: no voluntary movement; 1 point: barely perceptible movement; 2 points: frequent movement of hind limbs, no weight support; 3 points: alternate stepping or propulsive movement, no weight support; 4 points: hind limbs can support weight; 5 points: ambulation with mild deficit; and 6 points: normal ambulation.

In addition, we assessed the muscle strength in each group. Muscle strength was divided into five grades according to the Medical Research Council (MRC) scale for muscle strength (Compston, 2010): grade 5: normal muscle strength; grade 4: muscle strength is reduced, but muscle contraction can still move the joint against resistance; grade 3: muscle strength is further reduced such that the joint can be moved only against gravity with the examiner’s resistance completely removed; grade 2: muscle can move only if the resistance of gravity is removed; grade 1: only a trace or flicker of movement is seen or felt in the muscle or fasciculations are observed in the muscle; grade 0: no movement is observed.

### 2.6 Magnetic Resonance Imaging Examination

To get better MRI images of the central spinal cord lesions, we removed internal fixation in pigs. Pigs were maintained under isoflurane inhalation anesthesia throughout the examination. MRI of the porcine spinal cord was performed using a 3.0-T MRI scanner (Siemens, Berlin, Germany) 1 week after DSCI. Fifteen consecutive sagittal T2-weighted images were obtained by scanning the surgical regions of the pigs using a double-tuned volume radiofrequency coil. The parameters were set as follows: repetition time/echo time (TR/TE), 3500/103 ms; slice thickness, 3 mm; and slice gap, 10 percent. T2-weighted images were used to calculate the T2 intensity at the central DSCI lesion region (T15) using ImageJ software (National Institutes of Health, United States) (Yao et al., 2021).

### 2.7 Histological Staining

Seven days after DSCI, spinal cord samples were acquired after sacrifice, and the specimens were fixed in buffered formalin. After being immersed in 4% paraformaldehyde for 24 h and embedding in paraffin, a serial cross-section was made at the central DSCI lesion region. Hematoxylin and eosin (H&E) staining and Luxol fast blue (LFB) staining were used to evaluate morphological and structural changes in the white and gray matter, nerve sheath, and general spinal cord conditions in the pigs of different groups.

### 2.8 Enzyme-Linked Immunosorbent Assay

An enzyme-linked immunosorbent assay (ELISA) was carried out on samples from central DSCI lesions and cerebrospinal fluid (CSF) at 7 days after DSCI. Twenty milliliters of CSF were collected for ELISA before the pigs were sacrificed (Duan et al., 2018). After the spinal cord tissue was homogenized and centrifuged at 4000 rpm for 10 min, the liquid supernatant and cerebrospinal fluid were detected at 450 nm wavelength according to the manufacturer’s instructions for the IL-1β, IL-6, and TNF-α ELISA kits (Shanghai Jianglai Biological Technology Co., Ltd, China). The ELISA samples were run in triplicates.

### 2.9 Immunofluorescence Staining

After the pigs were sacrificed, spinal cord sections (1 cm) from the central DSCI lesion region were collected and embedded in paraffin. Spinal cord sections (5 μm thick) from each specimen were deparaffinized with xylene and incubated in graded concentrations of ethanol. They were then washed with phosphate-buffered saline (PBS) for 3 × 5 min. The sections were incubated for blocking with a blocking solution (0.1% Triton X-100 in PBS and 10% normal goat serum) at room temperature for 2 h. The sections were incubated overnight with primary antibodies at 4°C. The primary antibodies used were anti-GFAP (1: 2000, GeneTex), anti-Iba-1 (1:200, Affinity), and anti-NeuN (1:100, Abcam). After rinsing with PBS for 3 × 5 min, the sections were incubated with secondary antibodies for 2 h at room temperature. The secondary antibody used in this study was goat anti-rabbit antibody (Alexa Fluor^®^594) (1:200, Abcam). Following three rinses with PBS, a drop of antifade mounting medium containing DAPI (Solarbio Biotechnology, China) was placed on each slide. Finally, a coverslip was placed on top of each sample. Immunofluorescence imaging was carried out using an Olympus fluorescence microscope.

### 2.10 Western Blotting

The spinal cord tissues were removed from the liquid nitrogen, and the spinal cord was cut into small pieces and subsequently homogenized in radioimmunoprecipitation assay lysis buffer. The supernatant was obtained after centrifugation at a low temperature, and the protein concentration was determined using a bicinchoninic acid kit. Proteins were separated by gradient sodium dodecyl sulfate-polyacrylamide gel electrophoresis and electrophoretically transferred to polyvinylidene difluoride (PVDF) membranes. PVDF membranes were blocked with 5% non-fat dry milk for 2 hours and subsequently incubated with primary antibodies overnight at 4°C. The primary antibodies used were as follows: anti-Bcl-2 (1:2000, GeneTex), anti-Bax (1:1000, Biorbyt), anti-Caspase-3 (1:2000, Abcam), and anti-β-actin (1:5000, Proteintech). The washed membrane was incubated with goat anti-rabbit or goat anti-mouse IgG HRP-conjugated secondary antibody (1:5000). At last, PVDF membranes were exposed using a Tanon 5200 chemiluminescence image analysis system. Following exposure, ImageJ software (National Institutes of Health, United States) was used to analyze the gray values of bands.

### 2.11 Statistical Analysis

Statistical analyses were performed using GraphPad Software (United States). A student’s t-test was used to compare group means, and one-way analysis of variance (ANOVA) was used to compare multiple samples. Data are presented as the mean ± standard deviation (SD). Changes in muscle strength and modified Tarlov scores from baseline to day 7 were assessed with a generalized linear mixed model using PROC GLIMMIX in SPSS 22.0 (United States). A Bonferroni adjustment was carried out for multiple comparisons of the three groups. The interaction between time and degree of DSCI was also analyzed in the model. Statistical significance was considered *p < 0.05, **p < 0.01, and ***p < 0.001 vs. the sham group.

## 3 Results

### 3.1 Neurologic Function Changes of Hind Limbs Following Distraction Spinal Cord Injury

**FIGURE 2:**
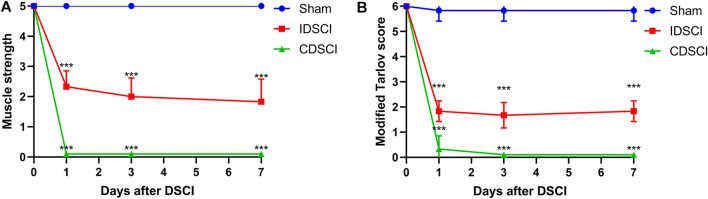
The neurologic function changes of hind limbs following DSCI. **(A)** The muscle strength of hind limbs at the different time points in the sham, IDSCI, and CDSCI groups. **(B)** The modified Tarlov score of the hind limbs at the different time points in the sham, IDSCI, and CDSCI groups. All values are expressed as means ± SD. *p < 0.05, **p < 0.01, ***p < 0.001 vs. the sham group.

As shown in Figure 2, the muscle strength of the hind limbs and the modified Tarlov score in the sham group maintained nearly normal at the 1, 3, 7 days after DSCI. At each time point, both the levels of muscle strength and modified Tarlov score in the two DSCI groups showed a significant decrease compared with the sham group (*p* < 0.001). Over time, the muscle strength of pigs in the IDSCI and CDSCI groups did not recover, and time was a factor influencing muscle strength in the two groups (F = 600.00; *p* < 0.001). For the two DSCI groups, muscle strength and modified Tarlov score of the CDSCI group reduced more significantly. ANOVA for repeated measures indicated significant differences among the three groups reciprocally in hind limb muscle strength (*p* < 0.001). Differences in the modified Tarlov score defects among the three groups were statistically significant. The modified Tarlov scores of the IDSCI and CDSCI groups did not improve over time (F = 852.76; *p* < 0.001).

### 3.2 DSCI Lesions Evaluated by MRI

**FIGURE 3:**
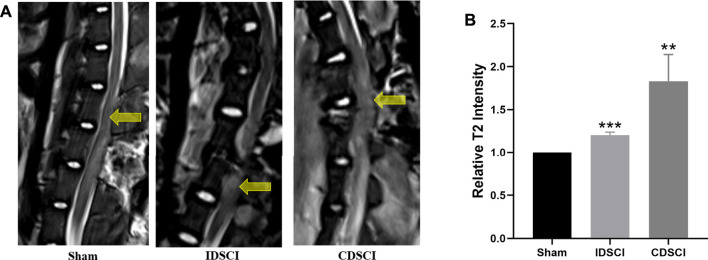
The DSCI lesions examined by MRI in different groups. **(A)** Representative T2-MRI images of the spinal cord in the sham, IDSCI, and CDSCI group. The central DSCI lesions were indicated by the yellow arrows. **(B)** The T2-MRI intensity in the central DSCI lesions was semi-quantified using ImageJ software. All values are expressed as means ± SD. *p < 0.05, **p < 0.01, ***p < 0.001 vs. the sham group.

We detected the imaging differences in the DSCI lesion area using MRI in live pigs of the three groups. As revealed in Figure 3A, T2-weighted MRI showed that hyperintense areas corresponded to central DSCI lesions areas of spinal cord in the IDSCI and CDSCI groups. Compared with the sham group, the increased enhancement in T2-weighted MRI was observed at the central DSCI lesions in the IDSCI (*p* < 0.001) and CDSCI groups (*p* < 0.01) (Figure 3B). And the relative intensity in T2-weighted MRI was higher in the CDSCI group with a higher degree of DSCI. The results may indicate that edema, inflammation, demyelination, axon loss, and astrogliosis occurred following DSCI.

### 3.3 Pathological Changes in the Spinal Cord Tissues Following Distraction Spinal Cord Injury

**FIGURE 4:**
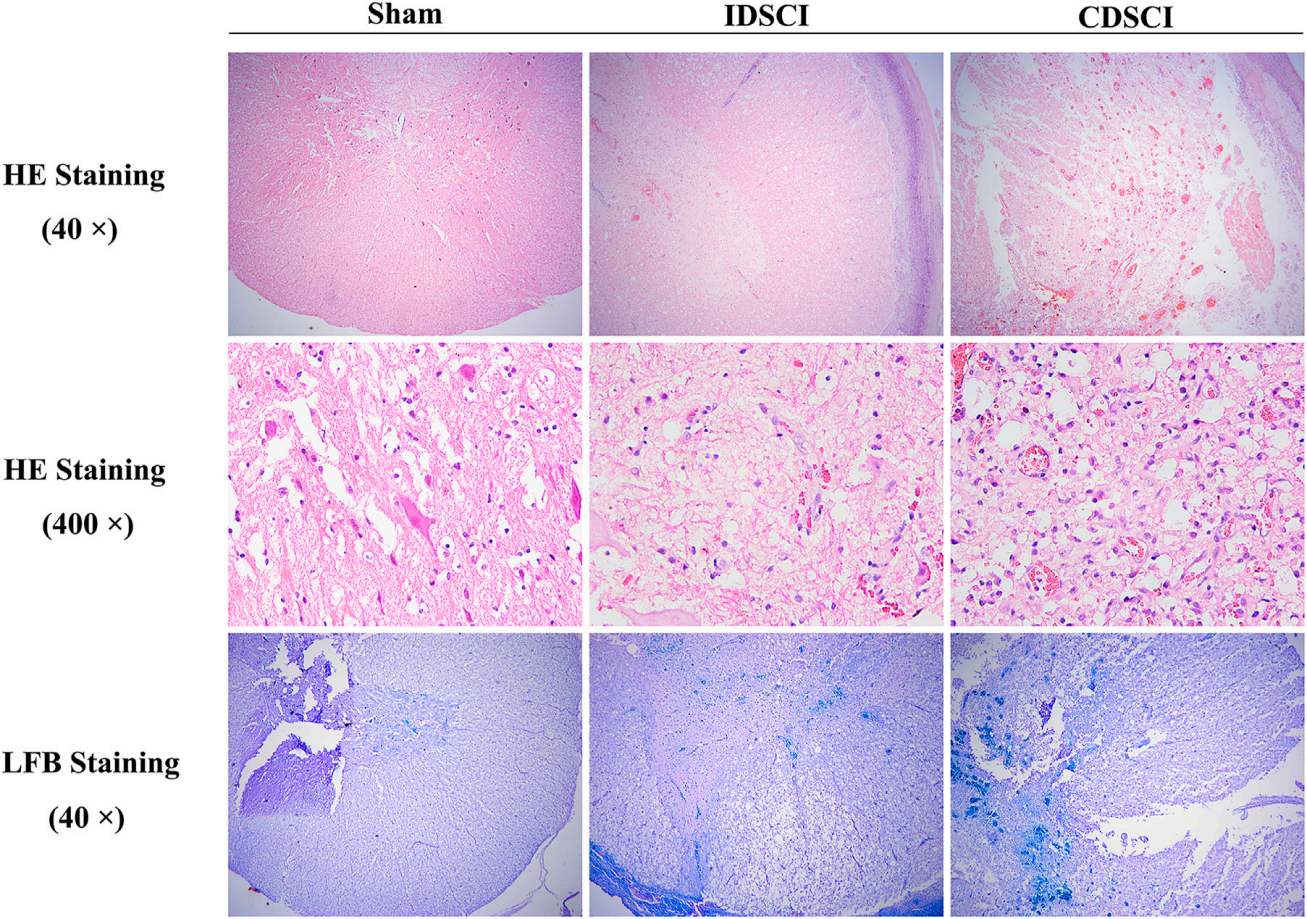
The pathological changes in the spinal cord tissues of different groups following DSCI. The representative images of spinal cord tissues stained with H&E (40 × and 400 ×) and LFB staining (40 ×) were shown.

As displayed in Figure 4, H&E staining showed a normal gray and white matter structure and normal neuronal morphology in the sham group. In the IDSCI group, it was revealed that damaged tissue structure, a decreased number of neurons, cell edema, and hyperemia in the spinal cord tissue. Injury to the spinal cord structure in the CDSCI group was more severe than that in the IDSCI group. The number of neurons decreased, hyperemia and edema were significant, alongside significant inflammatory cell infiltration.

Moreover, LFB staining stained myelin fiber deep blue. It was shown that a clear boundary differentiated from the surrounding structures in the sham group. The spinal tissue of IDSCI group showed disordered white matter arrangement, decreased density of nerve fiber bundles, and edema of the myelin sheath. In the CDSCI group, the white matter structure was further destroyed, and nerve fiber bundles showed a sparse network; in addition, myelin edema was obvious.

### 3.4 Levels of IL-1β, IL-6 and TNF-α in Spinal Cord Tissues and Cerebrospinal Fluid Following Distraction Spinal Cord Injury

**FIGURE 5:**
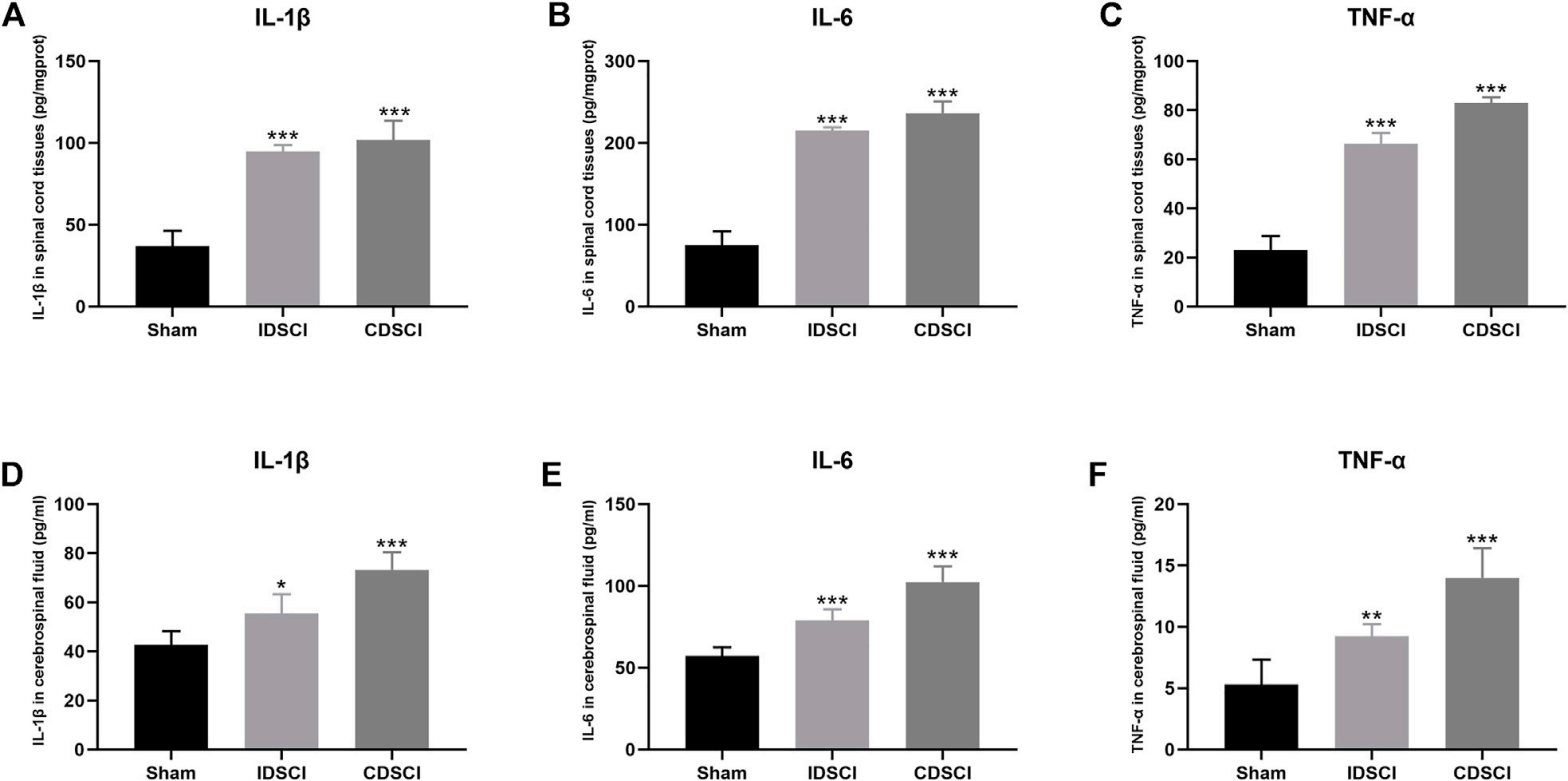
The levels of inflammatory cytokines in spinal cord tissues and cerebrospinal fluid at 7 days after DSCI. **(A–C)** The levels of IL-1β, IL-6 and TNF-α in spinal cord tissues of different groups. **(D–F)** The expression of IL-1β, IL-6, and TNF-α in CSF of different groups. All values are expressed as means ± SD. *p < 0.05, **p < 0.01, ***p < 0.001 vs. the sham group.

To detect the effect of different degrees of DSCI on the level of inflammatory cytokines in spinal cord tissues and CSF at 7 days after surgery, inflammatory cytokines were detected by ELISA (Figure 5). IL-1β, IL-6, and TNF-α levels in the two DSCI groups were significantly increased in spinal cord tissues after DSCI compared to the sham group (*p* < 0.001) (Figures 5A–C). Compared with the IDSCI group, the levels of IL-1β (*p* = 0.04), IL-6 (*p* = 0.03), and TNF-α (*p* < 0.001) were significantly higher in the CDSCI group.

Similarly, the levels of IL-1β, IL-6, and TNF-α in the CSF of the CDSCI group. These results indicate that the levels of inflammatory cytokines in CSF were elevated significantly in the two DSCI groups (*p* < 0.05) (Figures 5D–F). Among them, the concentration of IL-1β (*p* = 0.001), IL-6 (*p* < 0.001), and TNF-α (*p* = 0.002) were higher in the CSF of CDSCI group. These results indicate that the levels of inflammatory cytokines in spinal cord tissues and CSF increased with the increase in DSCI degree.

### 3.5 Neuroinflammatory Responses and Neuron Survival Maker Expression Detected by Immunofluorescence Staining in the Spinal Cord Lesions Following Distraction Spinal Cord Injury

**FIGURE 6:**
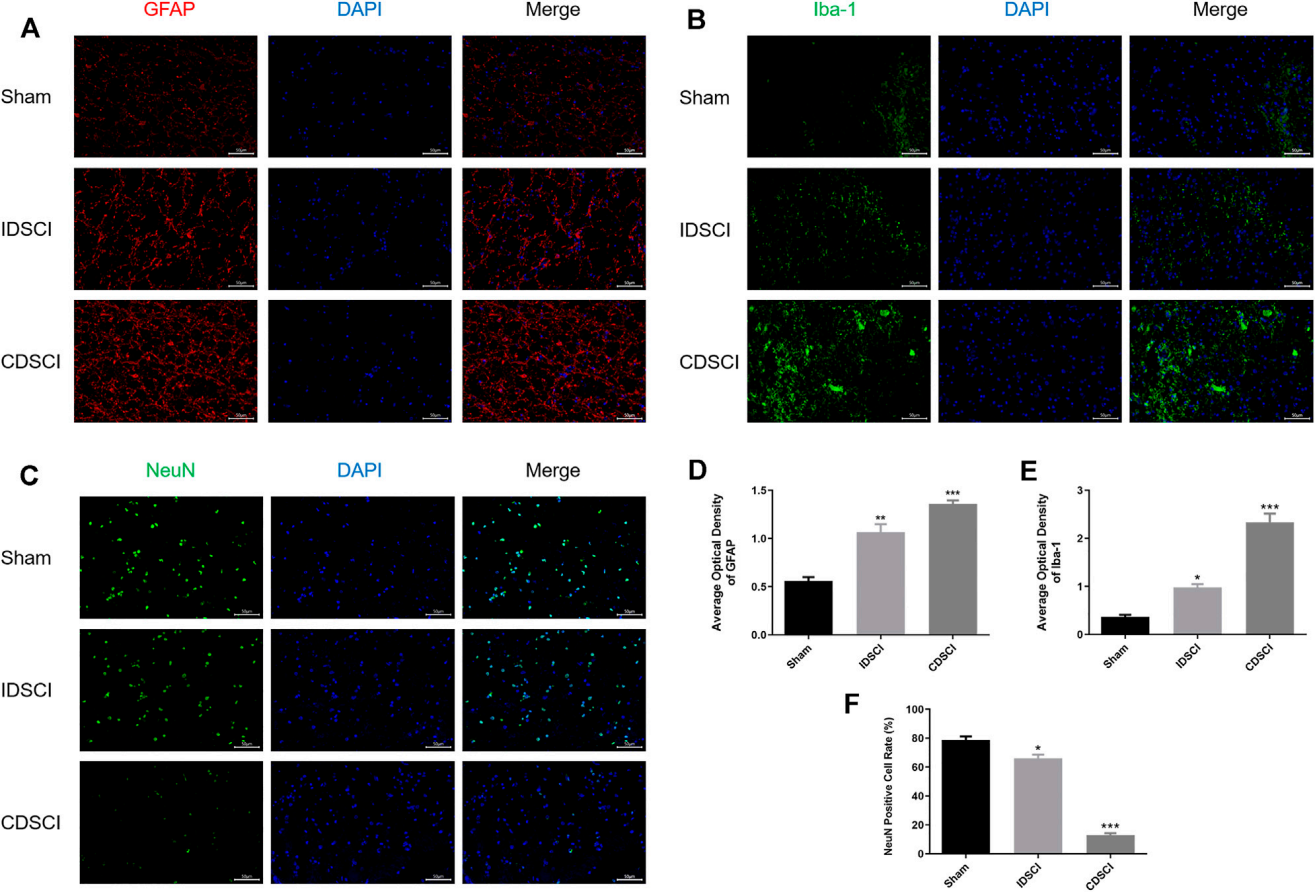
Immunofluorescence staining of GFAP, Iba-1, and NeuN in the central DSCI lesions at 7 days after DSCI in different groups. **(A)** Representative images (200 ×, scale bar = 50 μm) of GFAP (in red) and DAPI (in blue) staining. **(B)** Representative images (200 ×, scale bar = 50 μm) of Iba-1 (in green) and DAPI (in blue) staining. **(C)** Representative images (200 ×, scale bar = 50 μm) of NeuN (in green) and DAPI (in blue) staining. **(D,E)** The averaged optical density of GFAP, Iba-1, **(F)** and quantification of the NeuN-positive cells was measured (*n* = 3). All values are expressed as means ± SD. *p < 0.05, **p < 0.01, ***p < 0.001 vs. the sham group.

To assess the neuroinflammatory responses and neuron survival in the central DSCI lesions 7 days after DSCI, we conducted immunofluorescence staining to detect the expression levels of GFAP, Iba-1, and NeuN, as presented in Figure 6. We measured the expression of GFAP, a major component of scar matrices, to study the gliosis process after DSCI (Figure 6A). Iba-1, a major cell type involved in neuroinflammation, was also used to explore microglial activation (Figure 6B). GFAP and Iba-1 fluorescence was slightly expressed in the sham group. The optical density of GFAP and Iba-1 in the IDSCI and CDSCI groups was significantly increased compared with that in the sham group (*p* < 0.05) (Figures 6D, E). The optical density of GFAP and Iba-1 were higher in the CDSCI group than that in the IDSCI group. The number of GFAP-positive astrocytes increased with the DSCI degree increasing, pointing to gliosis hyperplasia. NeuN staining was used to assess neuronal survival after DSCI (Figure 6C). The expression level of NeuN in the IDSCI and CDSCI groups was significantly lower than that in the sham group (*p* < 0.05), and the CDSCI group presented a lower level between the two DSCI groups (Figure 6F).

### 3.6 Expression Levels of P53-Mediated Bcl-2/Bax/Caspase-3 Apoptosis Signaling Pathway-Related Proteins Following Distraction Spinal Cord Injury

**FIGURE 7:**
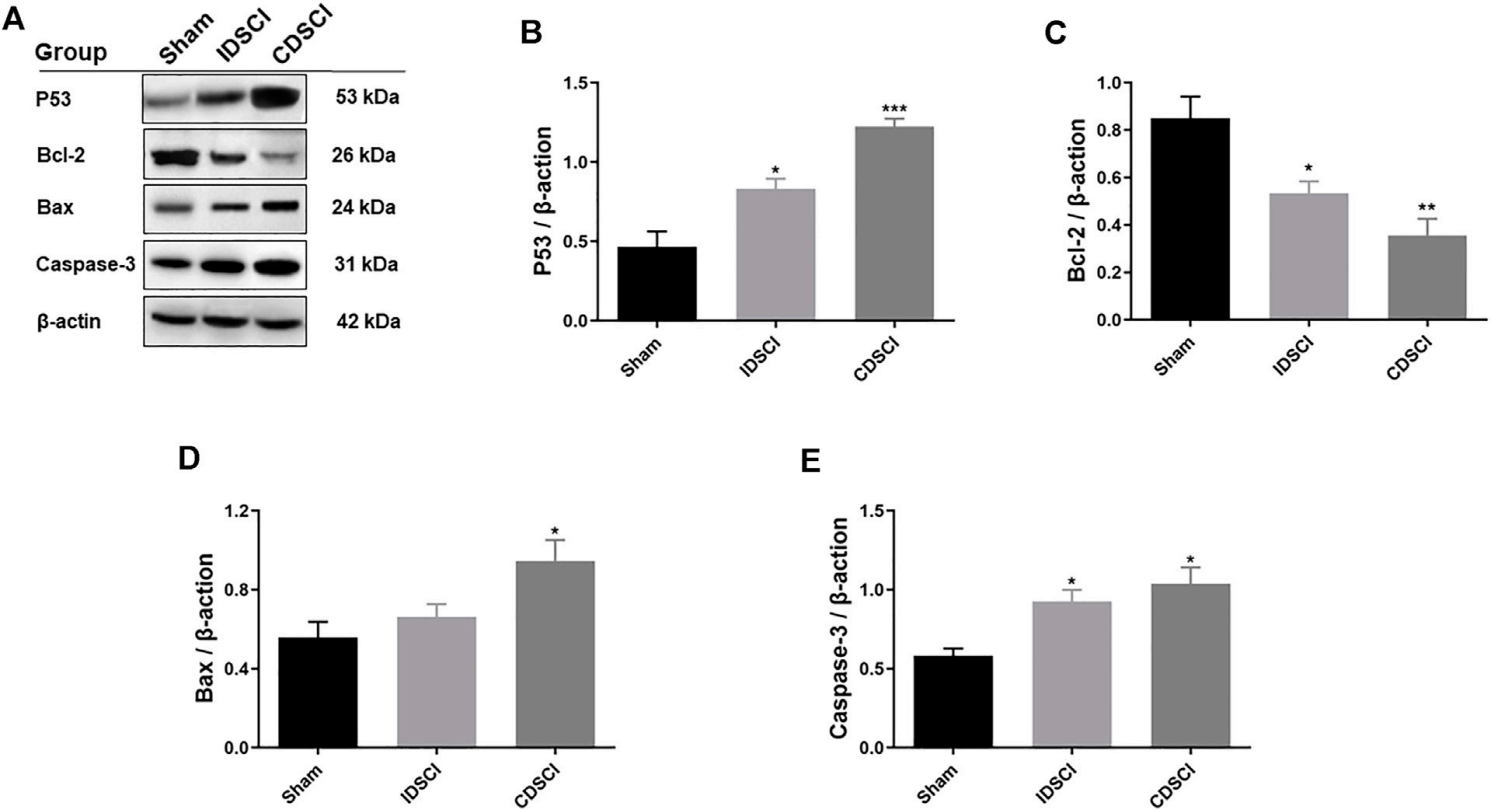
Determination of the expression levels of P53/Bcl-2/Bax/Caspase-3 signaling pathway-related proteins following DSCI. **(A)** The representative immunoblots of Bcl-2, Bax, Caspase-3, P53 and β-actin are listed. **(B–E)** Grayscale values were determined using ImageJ software based on the bands in the immunoblots (*n* = 3). All values are expressed as means ± SD. *p < 0.05, **p < 0.01, ***p < 0.001 vs. the sham group.

As represented in Figure 7, the expression levels of P53/Bcl-2/Bax/Caspase-3 apoptosis signaling pathway-related proteins were detected by western blotting. The results revealed that the expression levels of P53, Bax, and Caspase-3 proteins were significantly increased in the two DSCI groups (*p* < 0.05) except Bax protein in the IDSCI group, and the CDSCI group having a higher expression level. While Bcl-2 protein expression was significantly decreased in the two DSCI groups (*p* < 0.05), the decrease degree was more significant in the CDSCI group.

## 4 Discussion

DSCI is not uncommon in spinal surgery, and traumatic distraction has become a major cause of SCI, which was often accompanied by serious consequences such as complete paraplegia, incomplete paraplegia and neurological deficit. Most studies agree that there is always a potential risk of SCI during correction procedures by excessive spine distraction for spinal deformity (Heary et al., 2008; Pahys et al., 2009; Wu et al., 2016). The use of a growing rod for early-onset scoliosis increases the incidence of DSCI (Mattei et al., 2015). A review of 2209 SCI-related studies reported that 72.40% of SCI animal models were performed on rats (Cheriyan et al., 2014). However, due to the small size of the vertebrae in rats, pedicle screws cannot be inserted to simulate the distraction of the local spinal cord during surgery, as well as to mimic the changes in the spinal canal after osteotomy. Generally, DSCI models are established by stretching the spinal cord to simulate the tension forces experienced by the spinal cord in actual surgical SCI (Cheriyan et al., 2014). In the present study, we successfully established and verified a porcine DSCI model mimicking clinical DSCI, and preliminarily explored the role of microglial and astrocyte neuroinflammation and apoptosis in DSCI, providing potential strategies for DSCI treatment.

With regard to the model establishment approach of DSCI, computer-controlled stepping motor, distraction apparatus, and global column osteotomy with continuous distraction (GOCD) are the most common methods used in different animals (Chen et al., 2016; Wu et al., 2016; Bell et al., 2017; Wu et al., 2017; Shimizu et al., 2018; Tica et al., 2018; Guo et al., 2019; Wang J et al., 2019). From bench to bedside, severe spinal deformities can be treated by global column osteotomy techniques using an anterior, posterior, or a hybrid approach (Zhou et al., 2011). Moreover, GOCD mimics the process of osteotomy in spinal deformity correction surgery, which most likely leads to DSCI. In this study, GOCD was selected to establish the porcine DSCI models because it is close to various aspects of human DSCI.

The DSCI model was validated by T2-weighted MRI, neurologic function assessment of hind limbs, and histopathology examination in this study. T2-weighted MRI hyperintense areas may indicate the occurrence of edema, inflammation, demyelination, axon loss, and astrogliosis (Dalkilic et al., 2018). Compared with the sham group, in the two DSCI groups, the relative T2 hypodensity increased with the DSCI degree increasing, indicating that enhancement at the central DSCI lesions in T2-weighted MRI was more significant with the increase in the DSCI degree; further, edema, inflammation, demyelination, axon loss, and astrogliosis occurred after DSCI. Neurologic function results showed that porcine muscle strength and modified Tarlov score was significantly decreased after DSCI. Neurologic function defect of hind limbs was more severe in the CDSCI group with more serious DSCI. Histological staining of DSCI lesions was consistent with acute SCI pathologic changes shown as broken neural connective tissue, numerous vacuoles, and hemorrhagic infiltrations between the neural cells (Hong et al., 2016). These results indicated that GOCD could be used to establish the porcine DSCI model successfully. The GOGD method can be widely used for exploratory studies regarding the causes of, and potential treatment for, DSCI.

**FIGURE 8:**
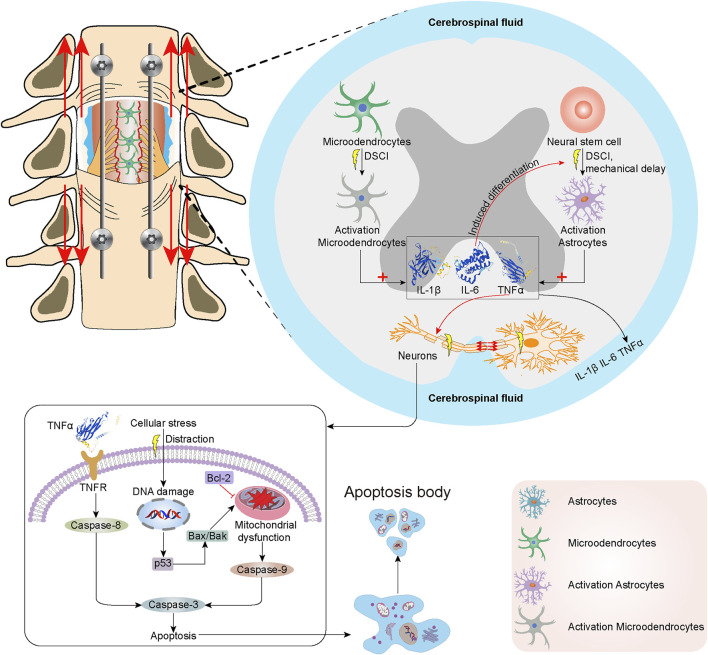
The mechanisms of neuroinflammation and cell apoptosis in the spinal cord following DSCI. DSCI induced acute activation of microglia, followed by delayed astrocyte activation. DSCI induces the acute activation of resident microglia and upregulation of inflammatory cytokines, including TNF-α, IL-1β, IL-6. Delayed astrocyte activation increases the expression of GFAP, which results in the production of multiple inflammatory cytokines. In extrinsic pathways, TNF-α binds to TNF-R1 on the surface of neurons to initiate apoptosis via Caspase-3 and Caspase-8. The intrinsic lethal stimuli induced by DSCI, such as the cellular stress, may cause DNA damage and upregulate the P53 expression. P53 induces Bax/Bak oligomerization in the mitochondrial outer membrane, which can promote the release of various intermembrane proteins. Bcl-2 suppresses Bax-induced apoptosis by forming a homodimer and heterodimer. Finally, Caspase-8, Caspase-9, Caspase-3 were activated to up-regulate their expression and initiate cell apoptosis of DSCI.

To our knowledge, this is the first report that discusses the potential molecular mechanisms underlying DSCI-associated microglial and astrocyte impairments. Based on our findings, the possible mechanisms of neuroinflammation and cell apoptosis in the spinal cord after DSCI are shown in Figure 8. The neuroinflammatory response is thought to play a pivotal role in secondary injury after SCI (Hohlfeld et al., 2007). DSCI induced acute activation of microglia, followed by delayed astrocyte activation. This study showed that the expression levels of TNF-α, IL-1β, IL-6, and Iba-1 in the DSCI lesions increased with the increase in the DSCI degree 7 days after injury, indicating that the occurrence of reactive gliosis and inflammation may be directly proportional to the degree of DSCI (Seifert et al., 2011).

Microglia, in a quiescent state under normal conditions, is the most common immune cell in the central nervous system (Greenhalgh et al., 2018). SCI induces the acute activation of resident microglia and upregulation of inflammatory cytokines, including TNF-α, IL-1β, IL-6, Iba-1, and iNOS (Saiwai et al., 2013). The GFAP expression in DSCI lesions increased with an increase in the degree of DSCI 7 days after injury. Astrocytes are immune effector cells that produce inflammatory cytokines (Duan et al., 2018). Delayed astrocyte activation increases the production of GFAP, which results in the production of multiple inflammatory cytokines (Yin et al., 2012). After the acute and subacute phases, the chronic phase is closely related to scar formation after injury and hinders nerve regeneration and repair (Li et al., 2019).

Overexpression of TNF-α, IL-1β, IL-6, Iba-1, and GFAP exacerbates inflammation and neurodegeneration (Hao et al., 2017). The inflammatory cytokines TNF-α and IL-1β can enhance vascular permeability (Mousavi et al., 2019). IL-6 regulates the inflammatory response and induces neural stem/progenitor cells to undergo astrocytic differentiation selectively, which is coordinated to hinder nerve repair after SCI (Mukaino et al., 2008). TNF-α, a mediator of cellular apoptosis, contribute to the apoptosis of oligodendrocytes in the spinal cord via the death domain of its cell surface receptor TNF-R1 (Cantarella et al., 2010). Microglial/macrophage activation and TNF-α and IL-6 overexpression in DSCI lesions causes oligodendrocyte necrosis and aggravates the damage to microglial cells (Yang et al., 2020).

In our study, NeuN staining revealed neuronal and oligodendrocyte death following DSCI. One of the primary causes of disability after DSCI is neuron damage and glial cell abnormity, which are not effectively replaced after injury. Cell death, mainly caused by the activation of apoptotic mechanisms during injury (Hurlbert et al., 2015), is a therapeutic target for DSCI treatment (Wang C et al., 2019). Apoptosis triggered by DSCI can be divided into extrinsic and intrinsic pathways. In extrinsic pathways, TNF-α binds to TNF-R1 on the surface of neurons to initiate apoptosis via Caspase-3 and Caspase-8 (Cantarella et al., 2010). DSCI induces the upregulation of death receptors and their ligands and the activation of caspases (Zhang et al., 2012). The intrinsic lethal stimuli of DSCI, such as the cellular stress by distraction, DNA damage and hypoxia, upregulates the expression of P53. P53 promotes the formation of BH3-only protein and induces Bax oligomerization in mitochondrial outer membrane, which can promote the release of various intermembrane proteins. P53 plays an essential role in regulating critical cellular processes, including cell cycle arrest and apoptosis. P53 overexpression has been reported to be occur during DSCI (Graham et al., 2020). Bcl-2 suppresses Bax-induced apoptosis of DSCI by forming a homodimer and heterodimer (Misgeld, 2005). Finally, Caspase-8 and Caspase-3 were activated to up-regulate their expression and initiate cell apoptosis of DSCI. Apoptosis is a programmed cell death process regulated by the signal transduction pathway; Bcl-2, Bax, and caspase-3 are the key proteins involved in apoptosis (Zhu et al., 2020). After DSCI, the proapoptotic proteins Bax and caspase-3 were upregulated, whereas the anti-apoptotic protein Bcl-2 was generally downregulated (Liu et al., 2015). In this study, we verified that apoptosis plays an important role in the progression of DSCI by determining the expression levels of P53-mediated Bcl-2/Bax/Caspase-3 apoptosis-related proteins. Our results were consistent with previous studies. The expression levels of P53, Bax, and Caspase-3 proteins promoting apoptosis increased significantly, while the expression of Bcl-2 protein that inhibits apoptosis decreased significantly in the two DSCI groups.

The limitations of this study are outlined as follows. At first, although the experimental objects of our study are large animals, our sample size is small. The mature modeling methods and molecular mechanisms provide a research basis for the DSCI experiments with large samples in the future. Secondly, due to the initial exploration of modeling methods and mechanisms of neuroinflammation and Apoptosis in DSCI, we did not carry out more intervention experiments. More interventions such as new synthetic and natural drugs and stem cell-derived exosomes will be conducted to identify optimal treatment options for DSCI.

## 5 Conclusion

In the present study, we successfully established porcine DSCI models with two different degrees of DSCI via GOCD. The DSCI models closely mimicked the mechanism of clinical DSCI and were used to elucidate the mechanisms underlying DSCI-associated neuroinflammation and apoptosis in DSCI. The neuroinflammation response after DSCI might be caused by the activation of microglia and astrocytes, which play a pivotal role in secondary injury after DSCI. The overexpression of IL-1β, IL-6, and TNF-α after DSCI may intensify the processes of inflammation and neurodegeneration. Moreover, the function and structure of impaired neurons and oligodendrocytes may be mediated by P53 mediated Bcl-2/Bax/Caspase-3 signaling pathway of apoptosis after DSCI. The neuroinflammation and apoptosis mechanisms presented in this study may provide potential therapeutic targets for DSCI in future research and therapy.

## Data Availability

The original contributions presented in the study are included in the article/supplementary material, further inquiries can be directed to the corresponding authors.
